# Indicators of haemodynamic instability and left ventricular function in a porcine model of esmolol induced negative inotropy

**DOI:** 10.1007/s10877-022-00937-8

**Published:** 2022-11-06

**Authors:** Simon Davies, Zhongping Jian, Feras Hatib, Amy Gomes, Monty Mythen

**Affiliations:** 1 Department of Anaesthesia, Critical Care and Perioperative Medicine, York Teaching Hospitals NHS Foundation Trust, York, UK; 2 Centre for Health and Population Science, Hull York Medical School, York, UK; 3 Edwards Lifesciences, Irvine, CA USA; 4 Hull York Medical School, York, UK; 5 UCL/UCLH National Institute of Health Research Biomedical Research Centre, London, UK

**Keywords:** Machine Learning, Haemodynamics, Instability, Negative inotropy

## Abstract

To investigate if the Hypotension Prediction Index was an early indicator of haemodynamic instability in a negative inotropy porcine model, and to assess the correlation of commonly measured indicators of left ventricular systolic function. Eight anaesthetised pigs were volume resuscitated and then underwent an incremental infusion of esmolol hydrochloride (0-3000 mg/hr), following which it was then reduced in a stepwise manner. Full haemodynamic measurements were taken at each stage and measurements of left ventricular systolic function including left ventricular stroke work index, ejection fraction and peripheral dP/dT were obtained. At an infusion rate of 500 mg/hr of esmolol there were no significant changes in any measured variables. At 1000 mg/hr MAP was on average 11 mmHg lower (95% CI 1 to 11 mmHg, *p* = 0.027) with a mean of 78 mmHg, HPI increased by 33 units (95% CI 4 to 62, *p* = 0.026) with a mean value of 63. No other parameters showed significant change from baseline values. Subsequent increases in esmolol showed changes in all parameters except SVV, SVR and PA mean. Correlation between dP/dt and LVSWI was 0.85 (95% CI 0.77 to 0.90, *p* < 0.001), between LVEF and dP/dt 0.39 (95% CI 0.18 to 0.57, *p* < 0.001), and between LSWI and LVEF 0.41 (95% CI 0.20 to 0.59, *p* < 0.001). In this model haemodynamic instability induced by negative inotropy was detected by the HPI algorithm prior to any clinically significant change in commonly measured variables. In addition, the peripheral measure of left ventricular contractility dP/dt correlates well with more established measurements of LV systolic function.

## Introduction

Major surgery and critical illness are associated with varying degrees of haemodynamic instability that may lead to hypotension and cardiac dysfunction, which are associated with adverse patient outcomes including mortality, acute kidney injury and myocardial injury [1–5]. Treatment of this instability frequently does not occur until there are marked changes in cardiovascular physiology such as a low mean arterial pressure or cardiac index [6], however events such as hypotension do not just physiologically occur but are preceded by changes in the compensatory mechanisms and other physiological signals that exist to maintain organ perfusion [7, 8]. Algorithms exist such as the Hypotension Prediction Index (HPI) that can measure the variability, complexity and physiological associations from a peripheral arterial waveform signal and can predict the future occurrence of hypotension [9]. It is derived from machine learning techniques based on features from a high fidelity arterial waveform, and displays a unitless number from 1 to 100, and the higher the number the more likely hypotension (defined as a MAP of < 65 mmHg for greater than one minute) is to occur in the future. This however may be an oversimplification of a complex algorithm that mathematically quantifies the magnitude of haemodynamic compensatory mechanisms, and hence the underlying degree of physiological instability within a biological system before any clinically relevant signs are manifested. Increasing output from the algorithm may represent increasing physiological instability which at a certain threshold will manifest itself as hypotension.

One potential cause of instability in the operative setting are the negative inotropic effects of anaesthetic agents, both intravenous [10–12] and inhalational [13, 14]. Left ventricular systolic function can be measured in various ways in the perioperative setting including the use of direct measurements such echocardiography, or indices of ventricular performance derived from a pulmonary artery catheter such as left ventricular stroke work index (LVSWI), however both echocardiography and pulmonary artery catheters are not routinely used in major non-cardiac surgery. Alternative measures of left ventricular function exist that can be derived from the peripheral arterial waveform. Peripheral arterial dP/dt_max_ (hereinafter dP/dt) has been shown to correlate with end systolic elastance, a load independent index of left ventricular function [15, 16], and LV dP/dt_max_ and can be measured continuously from an arterial signal, however it has not been validated against echocardiograph derived ejection fraction or LVSWI. Since HPI is a potential measure of physiological instability we hypothesised that esmolol induced negative inotropy would be represented by changes in HPI prior to clinically significant changes in other measured haemodynamic parameters. In addition, we aimed to determine the correlation of peripheral dP/dt with LVEF and LVSWI.

## Methods

The study was approved by the Institutional Animal Care and Use Committee (IACUC) at the Edwards Research Center, and all experimentation was performed in accordance with the USDA Animal Welfare Act regulations (AWArs), and the Guide for the Care and Use of Laboratory Animals (ILAR, NAP, Washington, DC, 2010, 8th edition). The Test Facility is accredited by the Association for the Assessment and Accreditation of Laboratory Animal Care, International (AAALACi) and registered with the United States Department of Agriculture to conduct research with laboratory animals. Relevant aspects of the ARRIVE (Animal Research: Reporting of In Vivo Experiments) guidelines were followed.

Eight female adult Yorkshire/ Landrace cross breed pigs with a mean weight of 79.7 (6.2) kg were studied. They were maintained in temperature and humidity-controlled rooms with a typical light–dark cycle and given standard food and tap water ad libitum. Prior to anaesthesia a general physical examination was performed to ensure all animal were in good health. Anaesthesia was induced with premedication using an intramuscular combination of telazol (4.4 mg.kg^−1^), ketamine (2.2 mg.kg^−1^) and xylazine (1.1 mg.kg^−1^). An endotracheal tube was then placed in the trachea, and anaesthesia maintained by 1–2% isoflurane in an oxygen and nitrous oxide mix (FiO_2_ 0.5). An intravenous catheter was placed in the auricular vein, and ECG and pulse oximetry established. Animals were ventilated on a volume-controlled mode with the respiratory rate set at 15 breaths per minute, and an initial tidal volume of 10 ml.kg^−1^ adjusted to maintain a PaCO_2_ of between 4.5 and 6 kPa. Positive end expiratory pressure was set at 5 cm H_2_O. Rectal temperature was monitored, and the animal temperature maintained between 38 and 40 °C using a heating pad.

Vascular access was then established under ultrasound guidance as follows; left femoral vein access was established with a 9F introducer sheath for fluid/drug administration, the right femoral vein was cannulated with an 8F sheath through which an intracardiac echo probe was introduced and a short axis view of the left ventricle obtained below the mitral valve. Measurements of left ventricular end diastolic diameter (LEDD) and end systolic diameter (LVESD) were taken and used to calculate left ventricular ejection fraction (LVEF) via the Teicholz formula [17]. The right femoral artery was cannulated with a 6F sheath for continuous pressure waveform measurements (FloTracIQ sensor; Edwards Lifesciences, Irvine, CA, USA) at a sampling rate of 100 Hz, using a HemoSphere monitor (Edwards Lifesciences, Irvine, CA, USA). This was used to record the following haemodynamic variables averaged at 20 s intervals; stroke volume (SV), heart rate (HR), cardiac output (CO), mean arterial blood pressure (MAP), systemic vascular resistance (SVR), dynamic arterial elastance (Eadyn), dP/dt and the hypotension prediction index (HPI). The left internal jugular vein was cannulated with an 8.5F triple lumen catheter for central venous pressure measurements (CVP) and the right internal jugular vein cannulated with a 9F introducer sheath through which a continuous cardiac output pulmonary artery flotation catheter was inserted into the pulmonary artery (Vigilance, Edwards Lifesciences, Irvine, CA, USA). The catheter was used to record continuous cardiac output (CCO), mean pulmonary artery pressure (MPAP), pulmonary artery occlusion pressure (PAOP) and mixed venous oxygen saturations (SvO_2_). Left ventricular stroke work index [18] was calculated via the equation SVI × (MAP–PAOP) × 0.0136.

The blood volume of the animals was calculated at 70 mL.kg^−1^. After induction of anaesthesia animals were infused with up to 10 mL.kg^−1^ of Lactated Ringers solution to achieve a SVV < 12%. Once this had been achieved if the MAP was less than 80 mmHg then an infusion of phenylephrine (100 mcg/mL) was commenced at 5 mL per hour and titrated to achieve a MAP > 80 mmHg. Once the desired targets had been achieved 10 min were allowed to ensure haemodynamic stability. This point was then defined as baseline and all measurements described above were recorded and at every subsequent time point. Following baseline measurements an infusion of esmolol hydrochloride (10 mg/mL) was started at 500 mg/hr and a period of 15 min allowed to achieve a steady state at which point all measurements were repeated (stage Esm 1). The infusion of esmolol hydrochloride was then subsequently increased to 1000 mg/hr, 2000 mg/hr and 3000 mg/hr with a similar period of steady state at each time point (stages Esm2, Esm 3 and Esm 4 respectively). Following measurements at stage Esm 4 the infusion rate was reduced to 2000 mg/hr (stage R1), 1000 mg/hr (stage R2) and then ceased (stage R3) allowing 15 min from dose reduction to measurements.

## Statistical analysis

Data are expressed as the mean (SD) or median (interquartile range) as appropriate. The normality of data distribution was checked using quantile–quantile plots. Differences at each experimental stage from baseline values were computed using a mixed effects model with the Geisser-Greenhouse correction and a Dunnet correction for multiple comparisons with individual variances computed for each comparison. Values are reported as a mean difference from baseline value (BA) with 95% confidence intervals. Correlation coefficients were calculated using Pearsons method. A P value of < 0.05 was considered statistically significant and all analysis was performed with GraphPad Prism version 9.3.1 (GraphPad software, La Jolla, California, US).

## Results

All animals received lactate ringers with the median dose being 500 mL (IQR 500 to 750), and 2 animals required phenylephrine infusions at a rate of 1500mcg/hr which was continued throughout the experiment.

**Fig. 1:**
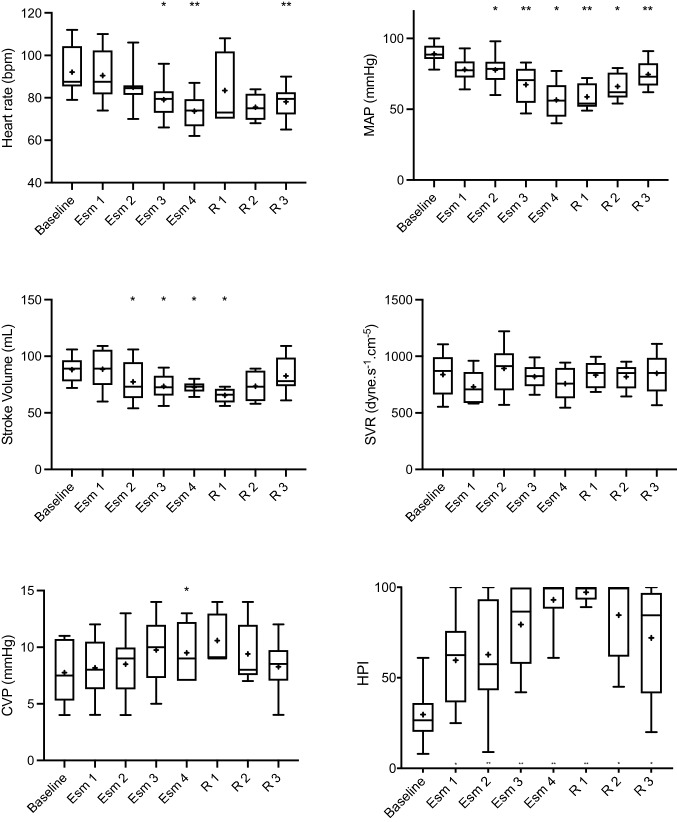
Box and whisker (5–95 percentile) plot of baseline values and changes in HR, MAP, SV, SVR, CVP and HPI over the different experimental stages. *P < 0.05, ^**^P < 0.01 compared to baseline values. + represents mean and solid line median

**Fig. 2:**
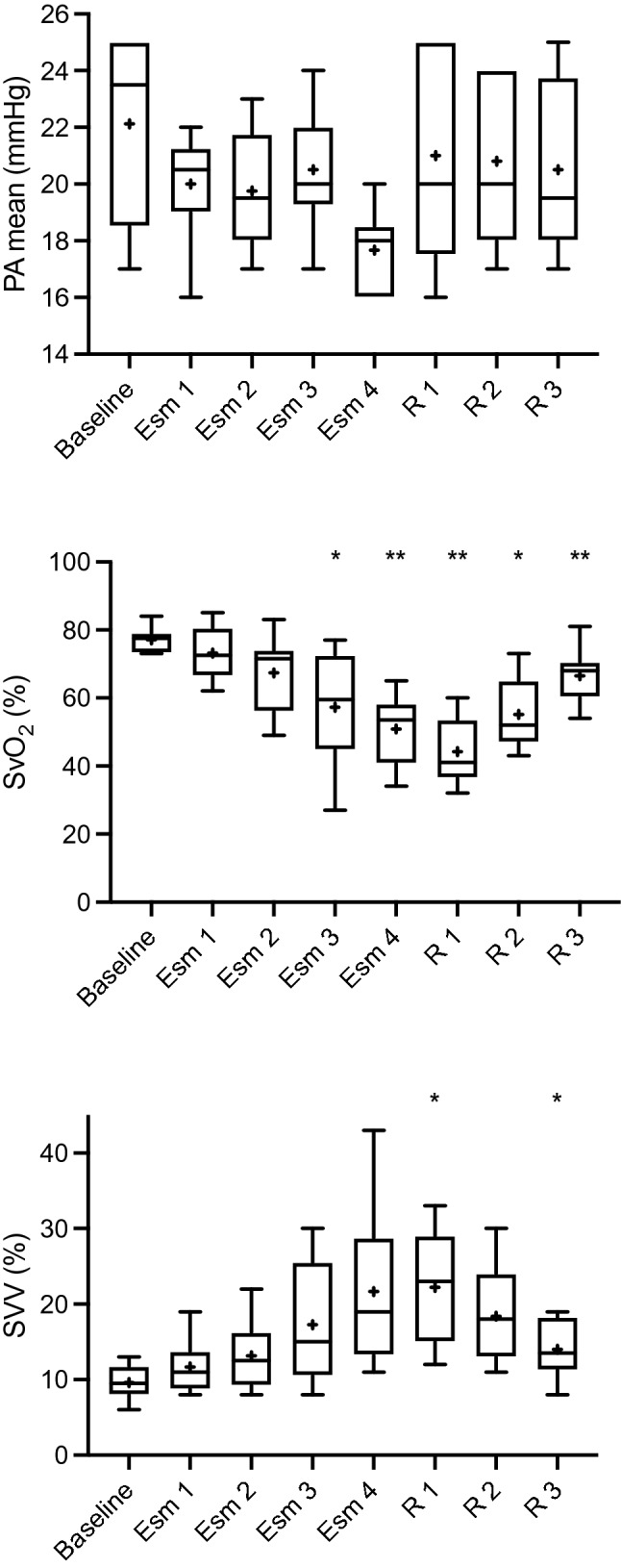
Box and whisker (5–95 percentile) plot of baseline values and changes in PA mean, SvO_2_ and SVV over the different experimental stages. *P < 0.05, ^**^P < 0.01 compared to baseline values. + represents mean and solid line median

Baseline haemodynamic variables and mean difference in values compared to baseline measurements at increasing doses of esmolol are shown in Table 1, and the absolute values are shown in Figs. 1 and 2.

**Table 1:** baseline mean values (SD) and mean difference (MD) with 95% CI for measured haemodynamic parameters compared to baseline values at each experimental stage. *P < 0.05, ^**^P < 0.01 compared to baseline values

	Baseline	Esm 1 (500 mg/hr)	Esm 2 (1000 mg/hr)	Esm 3 (2000 mg/hr)	Esm 4 (3000 mg/hr)
	Mean (SD)	MD (95% CI)	MD (95% CI)	MD (95% CI)	MD (95% CI)
Heart rate (bpm)	92(11)	−2 (−9 to 6)	−7 (−15 to 2)	−13 (−16 to 0)^⋆^	−18 (−27 to −9)^⋆⋆^
MAP (mmHg)	89 (7)	−11 (−23 to 1)	−11 (−21 to -1)^⋆^	−21 (−36 to−8) ^⋆⋆^	−33 (−54 to −11)^⋆^
Stroke Volume (mL)	88 (12)	0 (−17 to 18)	−10 (−27 to 6)	−-15 (−28 to −1)^⋆^	−16 (−30 to −1)^⋆^
Cardiac output (l/min)	8.1 (1.7)	0 (−2 to 2)	−1.5 (−3.7 to 0.8)	−2.3 (−4.3 to −0.2)^⋆^	−2.8 (−4.4 to −1.2) ^⋆⋆^
CVP (mmHg)	8 (3)	1 (−1 to 2)	1 (−1 to 2)	2 (−1 to 3)	2 (0 to 3)^⋆^
SvO_2_ (%)	77 (4)	−4 (−12 to 4)	−10 (−22 to 3)	−20 (−39 to −1)^⋆^	−26 (−41 to −12)^⋆⋆^
SVV (%)	10 (2)	2 (−2 to 6)	4 (−1 to 8)	8 (−1 to 16)	12 (−3 to 27)
dP/dt (mmHg.s^−1^)	434 (93)	−58 (−224 to -108)	−84 (−217 to 50)	−136 (−277 to 5)	−171 (−229 to −113)^⋆⋆^
LVSWI (g-m.m^−2^)	60 (11)	−6 (− 15 to 3)	−10 (−22 to 2)	−22 (−34 to −9)^⋆⋆^	−15 (−42 to 12)^⋆⋆^
LVEF (%)	75 (9)	−4 (−21 to 12)	−15 (−42 to 12)	−26 (−58 to 6)	−34 (−63 to −5)^⋆^
SVR (dyne.s^−1^.cm^−5^)	839 (194)	−109 (−291 to 73)	54 (−174 to 282)	−18 (−211 to 175)	−81 (−471 to 308)
PA mean (mmHg)	22 (3)	−2 (−8 to 4)	−2 (−6 to 1)	−2 (−5 to 2)	−4 (−9 to 0)
HPI	29 (16)	30 (−9 to 70)	33 (4 to 62)^⋆^	50 (20 to 80) ^⋆⋆^	64 (28 to 99)^⋆⋆^

	R 1 (2000 mg/hr)	R 2 (1000 mg/hr)	R 3 (0 mg/hr)
	MD (95% CI)	MD (95% CI)	MD (95% CI)
Heart rate (bpm)	−9 (−46 to 29)	−16 (−35 to 2)	−14 (−23 to −5)^⋆⋆^
MAP (mmHg)	−30 (−-47 to −13)^⋆⋆^	−23 (−41 to −5)^⋆^	−15 (−25 to −4)^⋆⋆^
Stroke volume (mL)	−22 (−40 to −5)^⋆^	−14 (−35 to 6)	−6 (−18 to 7)
Cardiac output (l/min)	−3.2 (−6.4 to −0.1)^⋆^	−2.5 (−5.6 to 0.5)	−1.6 (−3.2 to 0)^⋆^
CVP (mmHg)	3 (0 to 6)	2 (−1 to 4)	1 (−1 to 2)
SvO_2_ (%)	−33 (−48 to 17)^⋆⋆^	−22 (−38 to −5)^⋆^	−10 (−18 to −3)^⋆⋆^
SVV (%)	13 (2 to 23)^⋆^	9 (−1 to 19)	4 (0 to 9)⋆
dP/dt (mmHg.s^−1^)	−160 (−343 to 22)	−117 (−286 to 52)	−89 (−197 to 18)
LVSWI (g-m.m-^2^)	−42 (−60 to −23)^⋆⋆^	−35 (−53 to −17)^⋆⋆^	−28 (−38 to −17)^⋆⋆^
LVEF (%)	−23 (−56 to 9)	−16 (−49 to 17)	−17 (−40 to 6)
SVR (dyne.s^−1^.cm^−5^)	−5 (−481 to 472)	−19 (−435 to 397)	10 (−137 to 157)
PA mean (mmHg)	−1 (−7 to 5)	−1 (−7 to 5)	−2 (−5 to 2)
HPI	68 (37 to 98)^⋆⋆^	55 (8 to 100)^⋆^	42 (10 to 75)^⋆^

At an infusion rate of 500 mg/hr of esmolol (Esm 1) there were no significant changes in any measured physiological variables.

At an infusion rate of 1000 mg/hr of esmolol (Esm 2) MAP was on average 11 mmHg lower (95% CI 1 to 21 mmHg, *p* = 0.027) and a mean of 78 mmHg (SD 11). HPI increased by 33 units (95% CI 4 to 62, *p* = 0.026) with a mean value of 63 (SD 31). No other parameters showed significant change from baseline values.

At an infusion rate of 2000 mg/hr of esmolol (Esm 3) heart rate was 13 bpm lower (95% CI 0 to 16 bpm, *p* = 0.047) with a mean of 80 bpm (SD 9), MAP was 21 mmHg lower (95% CI 8 to 36 mmHg, *p* = 0.006) with a mean of 67 mmHg (SD 13), stroke volume had decreased by 15 mL (95% CI 1 to 28 mL, *p* = 0.034) with a mean of 73 mL (SD 11) with an associated decrease in cardiac output of 2.3 L/min (95% CI 0.2 to 4.3 L/min, *p* = 0.032). A 20% decrease in SvO_2_ occurred (95% CI 1 to 39%, p = 0.041) and LVSWI decreased by 22 g-m.m^−2^ (95% CI 9 to 34 g-m.m^−2^, *p* = 0.003). HPI was increased by 50 units compared to baseline (95% CI 20 to 80, *p* = 0.004) with a mean of 80 (SD 23).

After the final increase in infusion rate of esmolol to 3000 mg/hr (Esm 4) significant changes were seen in all parameters except for SVV, SVR and PA mean. Heart rate had decreased by 18 bpm (95% CI 9 to 27 bpm, *p* = 0.002) and stroke volume by 16 mL (95% CI 1 to 30 mL, *p* = 0.044) with a corresponding decrease in cardiac output of 2.8L/min (95% CI 1.2 to 4.4 L/min, *p* = 0.005) with a mean value of 5.3 L/min (SD 0.5). Mean arterial pressure had decreased by 33 mmHg (95% CI 11 to 54 mmHg, *p* = 0.01) to an average of 57 mmHg (SD 13). All measurements of left ventricular function showed a decrease at this stage with a 32 g-m.m^−2^ decrease in LVSWI (95% CI 15 to 50 g-m.m^−2^, *p* = 0.004), a 34% decrease in LVEF (95% CI 5 to 63%, *p* = 0.026) and a 171 mmHg.s^−1^ decrease in dP/dt (95% CI 113 to 229 mmHg.s^−1^, *p* < 0.001). HPI increased by 64 units (95% CI 28 to 99, *p* = 0.004) with a mean of 93 (SD15).

Mean difference in values compared to baseline measurements following reduction in esmolol infusion rates to 2000 and 1000 mg/hr respectively (R1 and R2) are shown in Table 1. After cessation of the infusion (R3) SV, CVP, SVR, dP/dt, LVEF and PA mean where not statistically different to baseline values. Heart rate was 14 bpm lower compared to baseline (95% CI 5 to 23 bpm, *p* = 0.007) with a mean of 78 bpm (SD 8), MAP was 15 mmHg lower (95% CI 4 to 25 mmHg, *p* = 0.009) with a mean 75 mmHg (SD 10) and SvO_2_ was 10% lower (95% CI 3 to 18%, *p* = 0.007). Cardiac output was 1.6 L/min lower than baseline (95% CI 0 to 3.3 L/min, *p* < 0.045) and LVSWI was 28 g-m.m^−2^ lower (95% CI 17 to 38 g-m.m^−2^, *p* < 0.001). HPI was 42 units higher compared to baseline (95% CI 10 to 75, *p* = 0.014).

### Correlation and changes in LVEF, dP/dt and LVSWI

**Fig. 3:**
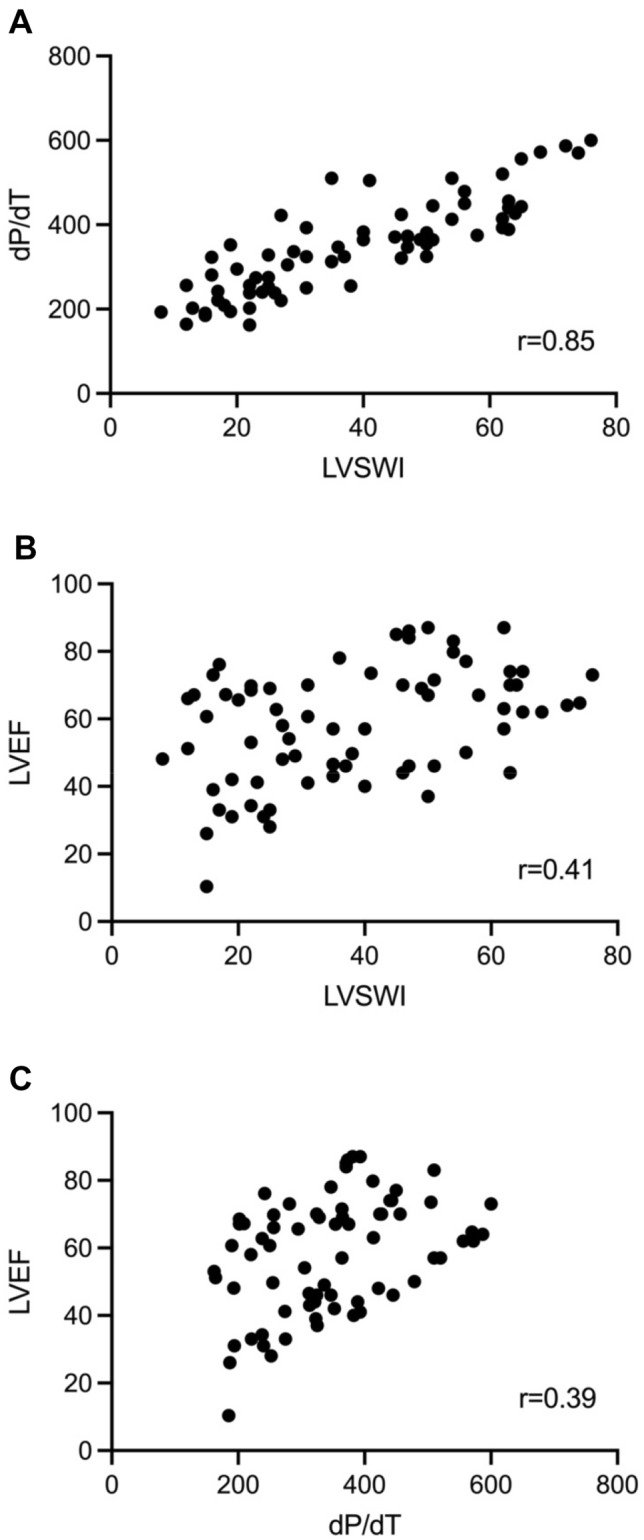
Correlation of LVEF, dP/dt and LVSWI

Correlation of LVEF, dP/dt and LVSWI are shown in Fig. 3. Correlation coefficient for dP/dt and LVSWI was 0.85 (95% CI 0.77 to 0.90, *p* < 0.001), and for LVEF and dP/dt the correlation coefficient was 0.39 (95% CI 0.18 to 0.57, *p* < 0.001), whilst for LSWI and LVEF the correlation coefficient was 0.41 (95% CI 0.20 to 0.59, *p* < 0.001).

**Fig. 4:**
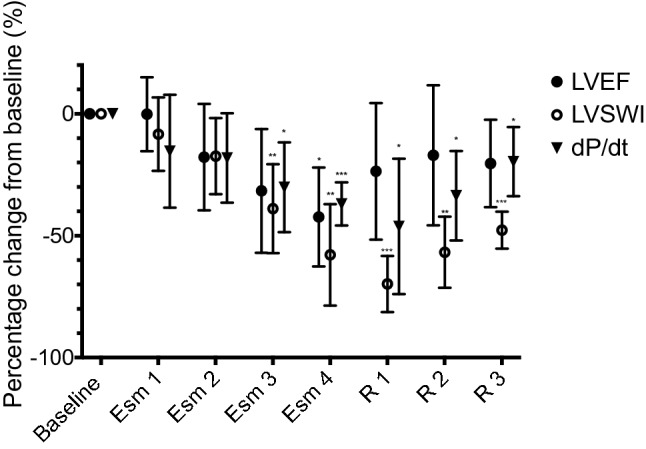
Percentage change from baseline values for LVEF, dP/dt and LVSWI over each experimental stage. *P < 0.05, ^**^P < 0.01, ^⋆⋆⋆^ *P* < 0.001 compared to baseline values

Percentage change from baseline values for LVEF, dP/dt and LVSWI are shown in Fig. 4. There was no significant percentage change from baseline in LVEF, dP/dt or LVSWI at stages Esm 1 and Esm 2. LVEF showed a significant change compared to baseline at stage Esm 4 only with a 42% decrease (95% CI 14 to 71, *p* = 0.01). dP/dt decreased by 30% at stage Esm 3 (95% CI 3 to 57, *p* = 0.03) and 37% at stage Esm 4 (95% CI 25 to 50, *p* = 0.001). Following cessation of the esmolol infusion values at R3 where not significantly different from baseline.

LVSWI decreased by 39% at stage Esm 3 (95% CI 12 to 65, *p* = 0.007) and 58% at stage Esm 4 (95% CI 29 to 87, *p* = 0.003). Following cessation of the esmolol infusion values at R3 LVSWI remained 48% lower than baseline values (95% CI 37 to 59, *p* < 0.001).

## Discussion

We have shown in this experimental study that the machine derived learning algorithm HPI detects instability induced by negative inotropy. This occurs prior to any clinically meaningful or significant change in any other measured haemodynamic variable. In addition, dP/dt, the arterial waveform derived measure of left ventricular function, showed a strong positive correlation with LVSWI.

The HPI was developed to predict the future occurrence of hypotension from features of the arterial waveform. Validation trials have shown the predictive ability of the algorithm to detect impending hypotension up to 15 min prior to the event. Several clinical trials have shown that the use of the technology in a protocolised manner can reduce the incidence and time weighted average of hypotension in subjects undergoing non-cardiac surgery [19, 20]. Most studies used a HPI threshold of either greater than 80 or 85 to initiate treatment. One trial in which use of the HPI did not reduce the amount of hypotension [21] used this threshold however noted the average time from warning to event was 4 min but in a quarter of patients the warning time was less than 2 min. Clinicians had little time to respond before hypotension occurred. Using a lower threshold provides a longer predictive time but increases the false positive rate [9], however it is this aspect that is of interest. Higher HPI thresholds are associated with higher event rates but a shorter time to event. For example, a threshold of between 80 and 89 has a 74.2% event rate with a median time to event of 3.74 min, compared to a threshold of between 50 and 59 has a lower event rate of 56.4% but a longer median time to event of 5.52 min. Using a lower threshold results in a higher false positive rate, but that is only if the event is classified as the binary outcome of hypotension.

‘Health’ may be thought of as a stable state compared to a disease process, however healthy biological processes show continual change and variation, whilst in an unstable state these variations are diminished. One example is variation in heart rate, where decreased variation is associated with both congestive heart failure and sudden cardiac death [22, 23]. These variations in biological parameters can be analysed using complexity theory to quantify these subtle changes over time. This essentially is what the HPI algorithm computes, the complexity in physiological measurements as well as the entropy (unpredictability in physiological measurements), the relationship between compensatory physiological processes in terms of baroreceptor sensitivity, variability, and spectral features amongst others. Hence HPI quantifies the amount of instability seen in a biological system, which may be thought of as the degree of compensatory mechanism required to maintain a ‘normal’ blood pressure. At higher degrees of compensation this will lead to hypotension, however if hypotension does not occur instability still remains. This this information may have clinical importance. In addition, if instability is described as magnitude of change from the healthy state baseline output, then there is the potential for treatment effectiveness to be quantified as the magnitude of change back towards the baseline.

This is reflected in the experimental data presented. At the lowest dose of esmolol infusion (500 mg/hr) the mean HPI increased from 30 to 60 but was not statistically significant and all other physiological parameters measured remained within normal limits. However, the magnitude of change at Esm 2 where HPI had a mean of 63 is similar to that seen at Esm 1. Whilst as mentioned this is not statistically significant, clinically this is likely to be interpreted in similar manner. If HPI is a marker of instability, then the threshold at which instability is deemed significant would need to be defined. At the rate of 1000 mg/hr HPI showed a statistically significant change with a mean value of 63, therefore this could be considered the initial threshold that represents haemodynamic instability, however further work would be needed to be able to define this with certainty. MAP was the only other parameter that showed a statistically significant change at Esm 2, however this remained within a normal range with a mean of 78 mmHg and would not be suggestive of instability in a clinical context.

Increasing doses of esmolol resulted in a statistically significant increase in HPI. Whilst other parameters showed significance at increased doses, many remained within normal limits and hypotension defined as a MAP < 65mHg only occurred at the maximal dose. This may suggest that HPI reflects the amount of instability conferred into the model by increasing infusion rates of esmolol, and is not simply a marker of impending hypotension. Whilst HPI is validated as being predictive of future hypotension at values of greater than 85, lower values may be suggestive of haemodynamic instability that may not necessarily lead to a hypotensive event due to activation of compensatory mechanisms. Other work supports the potential use of HPI as marker of instability. In a porcine model of haemorrhage at 5% volume loss HPI was significantly raised and associated with reduced intestinal microcirculation, whilst again no clinically significant changes were seen in other measured parameters [24].

If we can potentially use the algorithm as a marker of instability it would be useful to know whether the underlying cause is due to issue with preload, contractility or afterload. Esmolol is a cardioselective beta 1 adrenergic antagonist, and hence the mechanism of induced instability is through a decrease in contractility, heart rate and conduction [25]. It is reasonable to assume much of the instability was cause by negative inotropy. We compared three measures of left ventricular function namely LVEF, LVSWI and arterial dP/dt. Left ventricular stroke work index is dependent upon preload but afterload independent. It has been used in numerous studies as an indicator of altered contractility and relates to patient outcomes. Arterial dP/dt derived from an arterial waveform, however is influenced by preload and vascular filling conditions.

It correlates with end systolic elastance (Ees) and left ventricular dP/dt, although less accurate in underfilled conditions due to preload dependency [15]. Echocardiography derived LVEF is widely used in clinical practice, but has limitations as a measure of contractility due to its load dependency [26, 27], and is affected by ventricular geometry [28]. Afterload adjusted measurements have been suggested as a more accurate assessment of contractility [29]. None the less LVSWI and LVEF are used in clinical practice to assess contractility, and changes or trends in these values may be more useful than the absolute value. However, these measurements are often not available outside of the intensive unit, cardiac theatre or in those without a pulmonary artery catheter whose use is in decline. Arterial dP/dt provides a potential solution to allowing access to contractility information outside of these environments to guide treatment of instability. Arterial dP/dt correlated well with LVSWI, however whilst LVSWI and arterial dP/dt correlated with LVEF the relationship was less strong. It has been shown that LVSWI can be predictive of short and long term outcomes in patients on cardiac intensive care units [30]. If dP/dt can be used as a surrogate for LVSWI this has a potential advantage for easily measuring a parameter that confers prognostic information. We chose to use Teichholz method that has a number of limitations; however as we were interested in correlation rather than absolute agreement of values, and both LVSWI and arterial dP/dt had similar correlation coefficients.

There are several limitations to this study, firstly we chose to use esmolol as it is a cardio selective beta blocker, however it still has effects on peripheral vascular tone through nitric oxide mediated vasodilation so instability may be mixed in its aetiology especially at higher doses. This however is unlikely to significantly contribute to the instability seen early in the experiment. Secondly the use of the Teicholz method to calculate LVEF depends on geometric assumptions, and often overestimates LVEF. This method was chosen however as we were interested in showing correlation of LVEF with waveform derived measures of contractility rather than the absolute measurement. In addition, it is a simplistic measurement to calculate. The experiment was also performed on healthy porcine models, in whom it is reasonable to assume had no significant cardiovascular disease, and hence whether these observations will translate to human subjects with cardiovascular pathology where the technology is more likely to be used is unknown. Finally, although we have shown that HPI detects induced haemodynamic stability, the optimal value to define instability that has clinically significant implications has yet to be determined.

In this model haemodynamic instability induced by negative inotropy was detected by the HPI algorithm prior to any clinically significant change in commonly measured variables. In addition, the peripheral measure of left ventricular contractility dP/dt correlates well with more established measurements of LV function.
